# Psychotropics and the Microbiome: a Chamber of Secrets*…*

**DOI:** 10.1007/s00213-019-5185-8

**Published:** 2019-02-26

**Authors:** Sofia Cussotto, Gerard Clarke, Timothy G. Dinan, John F. Cryan

**Affiliations:** 1 APC Microbiome Ireland, University College Cork, Cork, Ireland; 2 Department of Anatomy and Neuroscience, University College Cork, Room 3.86, Western Gateway Building, Cork, Ireland; 3 Department of Psychiatry and Neurobehavioural Science, University College Cork, Cork, Ireland

**Keywords:** Psychotropic, Antipsychotic, Antidepressant, Antimicrobial, Gut microbiome

## Abstract

The human gut contains trillions of symbiotic bacteria that play a key role in programming different aspects of host physiology in health and disease. Psychotropic medications act on the central nervous system (CNS) and are used in the treatment of various psychiatric disorders. There is increasing emphasis on the bidirectional interaction between drugs and the gut microbiome. An expanding body of evidence supports the notion that microbes can metabolise drugs and vice versa drugs can modify the gut microbiota composition. In this review, we will first give a comprehensive introduction about this bidirectional interaction, then we will take into consideration different classes of psychotropics including antipsychotics, antidepressants, antianxiety drugs, anticonvulsants/mood stabilisers, opioid analgesics, drugs of abuse, alcohol, nicotine and xanthines. The varying effects of these widely used medications on microorganisms are becoming apparent from in vivo and in vitro studies. This has important implications for the future of psychopharmacology pipelines that will routinely need to consider the host microbiome during drug discovery and development.

## Introduction

In the second instalment of J.K. Rowling’s Harry Potter book series, the trainee wizards encounter a magical *Chamber of Secrets* deep within the *Hogwarts School*. In some ways, this story parallels the human body, with the gastrointestinal tract hidden within and guarding many secrets.

The human gastrointestinal tract harbours trillions of microbes, the gut microbiota, which help modulate developmental, immunological and nutritional function in the host (Bengmark 2013; Sampson and Mazmanian 2015; Soto et al. 2018; Valdes et al. 2018; Wang et al. 2016). The colonisation of the gut is generally believed to begin at birth with the infant initially receiving microbial colonisation from the mother as it passes through the birth canal, although this notion has been challenged by a limited number of studies in which microbes were detected in the placenta (Aagaard et al. 2014; Collado et al. 2016; DiGiulio 2012). In a recent review paper, a critical assessment of the evidence supporting these two opposing hypotheses has been carried out and the authors argue that the evidence in support of the “in utero colonization hypothesis” is conceptually and materially flawed (Perez-Muñoz et al. 2017). Events such as illness, antibiotic treatment and changes in diet cause shifts in the microbiota (Codagnone et al. 2018; Conlon and Bird 2015; De Filippo et al. 2010; Rodrigues et al. 2017; Wang et al. 2017). Mode of delivery at birth also affects the microbiota composition, with vaginally delivered infants containing a high abundance of lactobacilli during the first few days, a reflection of the high load of lactobacilli in the vaginal flora (Aagaard et al. 2012; Avershina et al. 2014). In early stages of development, the microbiota is generally low in diversity and is dominated by two main phyla, Actinobacteria and Proteobacteria (Rodriguez et al. 2015). During the first year of life, the microbial diversity increases and by around 2.5 years of age, the composition, diversity and functional capabilities of the infant microbiota resemble those of an adult microbiota (Koenig et al. 2011; Rodriguez et al. 2015). In individuals over the age of 65, the microbial community changes, with an increased abundance of Bacteroidetes phyla and Clostridium cluster IV, in contrast with younger subjects where the cluster XIVa is more prevalent (Claesson et al. 2011). It has been demonstrated that the microbiota of young adults and 70-year-old people is highly similar but differs significantly from that of centenarians (Biagi et al. 2010).

The role of the microbiota in health and disease has stretched to all disciplines of medicine and this now includes pharmacology and therapeutics (Walsh et al. 2018). The field of pharmacomicrobiomics has emerged over the past decade (ElRakaiby et al. 2014; Saad et al. 2012) and has predominantly focused on the impact that the gut microbiota exerts on drug metabolism. Also, a growing body of research has demonstrated that several pharmaceutical compounds, including paracetamol, digoxin, metformin and cancer drugs among others, influence the human gut microbiota and/or microbial isolated strains. As bacteria can, in turn, modulate drug efficacy and toxicity (Alexander et al. 2017; Currò 2018; Li et al. 2016), the emerging drug-microbe bidirectional interaction might be crucial for future drug development and clinical practice. Moreover, this suggests that drug-related confounding effects should be taken into consideration in future microbiome association studies.

**Fig. 1:**
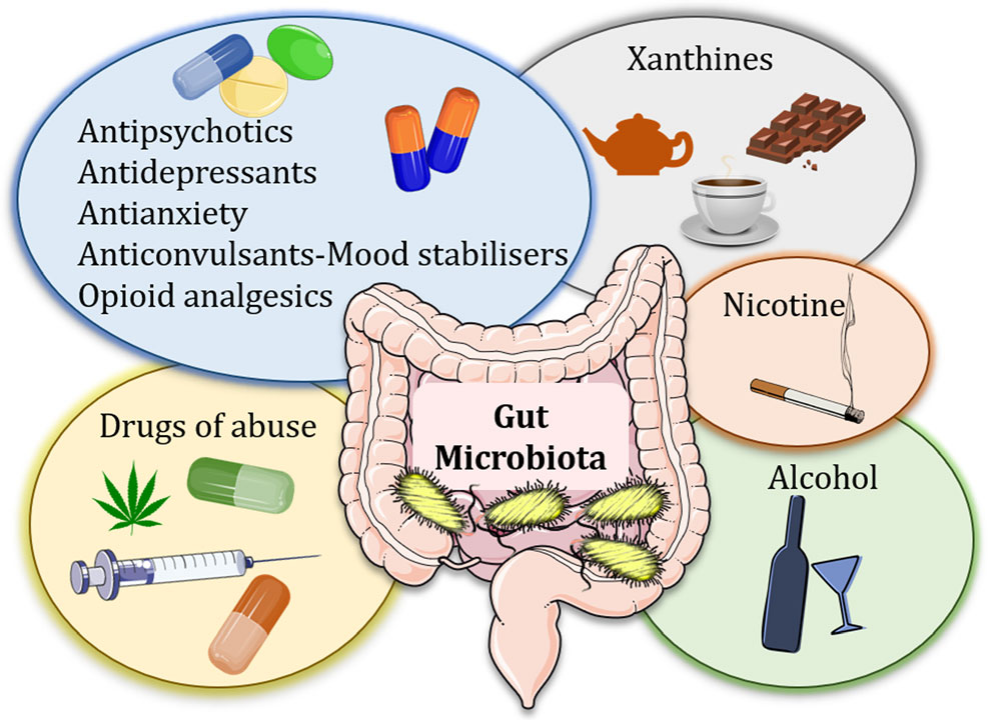
Psychotropic compounds affect the gut microbiota composition

In this review, we will focus on psychotropic compounds (from the Greek root *psychè* = mind and *tropòs* = turning), which modulate brain and behaviour, and we will explore the scientific evidence on the interaction between psychotropic compounds and the gut microbiome in vivo or in isolated strains (in vitro*)* (Fig. 1). For each class of psychotropic compound taken into consideration, sub-sections will be based on the experimental approach used (observations in vitro, in vivo or in humans). Regarding in vitro experiments, some attempts have been made to try and find the best dose translational to the human gut setting. Maier and colleagues have deduced colon concentrations on the basis of drug excretion patterns from published work and small intestine concentrations on the basis of daily doses of individual drugs. Based on their approximations, a threshold of 20 μM was below the median small intestine and colon concentration of the majority of human-targeted drugs (Maier et al. 2018). It is important to keep this in mind when considering data generated from in vitro isolated microbial strains.

The literature search (PubMed) was conducted using the following terms as inclusion criteria: the chemical name of each drug belonging to either of the following classes: antipsychotics, antidepressants, antianxiety drugs, anticonvulsants/mood stabilisers, opioid analgesics, drugs of abuse OR alcohol/ethanol OR nicotine OR xanthines (caffeine, theobromine, theophylline) AND (gut microbiome OR gut microbiota OR antimicrobial) up to 20 January 2019. Reviews, meta-analyses and systematic reviews were omitted from the search strategy.

## Drugs affect the gut microbiota

In Rowling’s book, the trainee wizards eventually find the gate to the *Chamber of Secrets*; similarly, xenobiotic compounds belonging to several therapeutic classes can reach the *Chamber* and affect what is hidden within, our gut microbiota.

Antibiotics represent the most direct and effective way of targeting intestinal microbes. Evidence gathered from in vitro and in vivo studies suggests that a course of short-term antibiotics can substantially change the gut microbiota composition (Jakobsson et al. 2010; Maurice et al. 2013). Several host-targeting non-antibiotic drugs have also been shown to influence the gut microbiota. In a population-based cohort, deep sequencing of gut microbiomes of 1135 participants showed relations between the microbiota and 19 drug groups (Zhernakova et al. 2016). Other studies have pointed out an association between drug consumption and microbiome. Analysis of two independent population-level cohorts revealed that, among different factors, the use of medications was responsible for the largest total variance and interacted with other covariate-microbiota associations (Falony et al. 2016). The composition of the gut microbiota can change in relation to the number and type of medications consumed. Differences in the relative abundance of specific bacteria were detected in individuals taking a single drug, a combination or none. In particular, there were differences in the gut microbiota of individuals taking NSAIDs (non-steroidal anti-inflammatory drugs) with PPIs (proton-pump inhibitors) versus those taking NSAIDs without PPIs (Rogers and Aronoff 2016). Regarding polypharmacy, in elderly hospitalised patients, there was a significant negative correlation between the number of drugs and microbial alpha-diversity (Chao1 index). Moreover, the number of drugs was associated with the average relative abundance of 15 different taxa, with PPIs, antidepressants and antipsychotics exhibiting the strongest association with single bacteria abundance (Ticinesi et al. 2017).

## The gut microbiota affects the pharmacokinetics of drugs

According to the novel, the *Chamber of Secrets* is home of a giant serpent, the *basilisk*. Similar to the *basilisk*, which has the ability to kill by looking people in the eyes, the microbes harboured in our guts can influence to a certain extent the pharmacokinetics of drugs.

Pharmacokinetics (from the Greek root *pharmakon* = drug and *kinetikos* = moving, “putting in motion”) is a branch of pharmacology dedicated to determining the fate of xenobiotics administered to a living organism. Absorption is one of the four compartments of the pharmacokinetics multi-compartmental model (Arundel 1997), together with distribution, metabolism and excretion (ADME) (Pacey et al. 2011).

In the next two sections, we provide some of the most compelling evidence on the interaction between gut microbiome and drug absorption/metabolism prior to discussing the relevance to psychotropic compounds.

### The gut microbiota affects drug absorption

In pharmacology, absorption is the movement of a substance from the site of administration to the bloodstream (Doogue and Polasek 2013). Very little is currently known about the role played by the gut microbiota in drug absorption but a few reports on the topic exist. It is interesting to note that all the three studies mentioned in this section use the same experimental approach: manipulation of the gut microbiota through administration of probiotics.

The action of **gliclazide**, a sulfonylurea used to treat diabetes, may be enhanced by administering probiotics. In diabetic rats, the blood levels of gliclazide are higher following a 3-day pre-treatment with probiotics (at the dose of 75 mg/kg) compared to non-treated rats, suggesting that the gut microbiota might mediate the extent of the drug absorption (Al-Salami et al. 2008). In a recent study, a 3-day administration of *Lactobacillus reuteri* K8 reduced the absorption of orally administered **acetaminophen** in mice, whereas administration of *Lactobacillus reuteri* K9 did not have an effect (Kim et al. 2018). This effect was probably mediated by probiotic-induced modulation of gut microbial enzyme activity given that the probiotic significantly increased both sulfatase and arylsulfate transferase and significantly decreased β-glucuronidase, which are the bacterial enzymes involved in acetaminophen metabolism. Finally, the antiarrhythmic drug **amiodarone** shows elevated blood levels following administration of probiotics in rats. In details, the probiotic *E. coli* strain Nissile 1917 was administered to rats for 7 days, followed by a single dose of amiodarone per os. The probiotic increased amiodarone plasmatic levels by 43% compared to saline-treated controls, suggesting a microbiota-mediated increase in drug absorption (Matuskova et al. 2014).

### The gut microbiota affects drug metabolism

The fate of xenobiotics depends not only by the host but also by the bacteria harbouring our gastrointestinal tract and it has become more investigated, over the past decades, the role of gut microbiome in xenobiotic metabolism. The whole field has been termed “pharmacomicrobiomics” (Rizkallah et al. 2010). In this paragraph, we offer a glimpse into the known effects of the gut microbiota on drug metabolism.

**Digoxin**, a cardiac glycoside that has been widely used for hundreds of years to treat heart failure and arrhythmias, is a striking example. This drug is inactivated in the gut by the Actinobacterium *Eggerthella lenta* (Haiser et al. 2013). Moreover, increased consumption of dietary protein in germ-free mice inhibited the reduction of digoxin by *E. lenta* (Haiser et al. 2013). The microbial biotransformation of orally administered **lovastatin**, a drug used for lowering cholesterol levels and reduce the risk of cardiovascular disease, was reduced by concomitant administration of antibiotics in rats (Yoo et al. 2014). This could result in altered systemic concentrations of either the intact drug and/or its metabolites (Yoo et al. 2014). **Amlodipine**, a medication used to treat high blood pressure and coronary artery disease, undergoes clearance when incubated with a faecal suspension, suggesting that the gut microbiota metabolises this drug (Yoo et al. 2016). As a confirmation, a 2-day treatment with the antibiotic ampicillin in rats increases the plasma levels of amlodipine, possibly because of the decreased microbial biotransformation in the gastrointestinal tract (Yoo et al. 2016). **Mesalazine**, also known as 5-aminosalicylic acid (5-ASA), is an anti-inflammatory drug used to treat inflammatory bowel disease, including ulcerative colitis or to maintain remission in Crohn’s disease (Rachmilewitz 1989). The faecal microbiota plays a key role in acetylating 5-ASA, with 44% of anaerobic bacteria tested in incubation with the drug exhibiting this property (van Hogezand et al. 1992). The metabolism of **sulfasalazine**, a drug used for the treatment of rheumatoid arthritis, ulcerative colitis and Crohn’s disease, is also likely to be mediated by intestinal bacteria. When sulfasalazine was fed to conventional rats, none of the drug was recovered in the urine, faeces or caecum; however, if administered in combination with the antibiotic neomycin, the drug was recovered in faeces and the caecum (Peppercorn and Goldman 1972). In addition, when sulfasalazine was given to germ-free rats, recovery of drug in the faeces was over 50% whereas the urine contained an additional 1–2%. In germ-free rats infected with four specific bacteria normally found in the intestinal tract of rodents, sulfasalazine was metabolised as in conventional rats (Peppercorn and Goldman 1972). Sulfasalazine is metabolised by azoreductases in the gut. The probiotic strains *Lactobacillus acidophilus* L10, *Bifidobacterium lactis* B94 and *Streptococcus salivarius* K12 given to rats for three consecutive days increased azoreductase activity in ex vivo colon contents with a corresponding increase in sulfasalazine metabolism (Lee et al. 2012). Interestingly, however, the same probiotic treatment in rats, followed by an oral 100 mg/kg dose of sulfasalazine, did not alter the pharmacokinetic parameters (Lee et al. 2012). Administration of **diclofenac**, a nonsteroidal anti-inflammatory drug (NSAID), induced enteropathy in mice; however, oral pre-treatment with a bacteria-specific β-glucuronidase inhibitor was able to protect against diclofenac-induced enteropathy (LoGuidice et al. 2012), suggesting that the gut microbiota might play a crucial role in the metabolism of this medication. The antithrombotic effect of **aspirin** seems to be affected by the gut microbiota. In rats, administration of the antibiotic ampicillin significantly prolongs the bleeding time in aspirin-dosed rats (Kim et al. 2016). Moreover, oral administration of ampicillin reduces the aspirin-metabolising activity of the microbiota by 67% (Kim et al. 2016).

Intestinal microbial azoreductases play a key role in the reduction of **azo dyes** (Chung et al. 1992). A wide variety of anaerobic bacteria isolated from caecal or faecal contents from experimental animals and humans have the ability to cleave the azo linkages to produce aromatic amines (Chung et al. 1992). Moreover, the azoreductase activity in a variety of intestinal preparations is affected by various dietary factors including antibiotics and supplementation with live cultures of lactobacilli (Chung et al. 1992).

**Choline** and **carnitine** are dietary amines that have wide-ranging roles in human metabolism (Zeisel and da Costa 2009) and are precursors of trimethylamine (TMA), a compound that can cause trimethylaminuria when not appropriately metabolised by the host (Mackay et al. 2011). In a recent study, the quantification and detailed characterisation of the TMA-producing bacteria in human faecal samples have resulted particularly in *Clostridium* XIVa strains and *Eubacterium* sp. strain AB3007 (Rath et al. 2017). In a different study, carnitine metabolism was mediated by Rieske-type oxygenases present in the human microbiota (Zhu et al. 2014).

**Chemotherapeutic drugs** have also been shown to be metabolised by the gut microbiota (Alexander et al. 2017). Of 30 chemotherapeutic drugs examined in vitro, the efficacy of 10 was found to be significantly inhibited by certain bacteria, while the same bacteria improved the efficacy of six others (Lehouritis et al. 2015). As further corroboration of these findings, the chemoresistance or increased cytotoxicity observed in vitro with sample drugs (gemcitabine and CB1954) was replicated in in vivo murine subcutaneous tumour models (Lehouritis et al. 2015). The dose-limiting side effect of the common colon cancer chemotherapeutic **irinotecan** is severe diarrhoea that arises following reactivation of the drug by symbiotic bacterial β-glucuronidases in the gut (Ma and McLeod 2003; Mathijssen et al. 2001). Oral administration of a bacterial β-glucuronidase inhibitor protected mice from irinotecan-induced toxicity, suggesting that such inhibitors may be designed to prevent undesirable enzyme activities in the intestine (Wallace et al. 2010). The gut microbiota also plays a crucial role in the metabolism of **5-fluorouracil**, another chemotherapeutic compound (Nakayama et al. 1997). The antineoplastic drug **doxorubicin** is effectively metabolised by *Raoultella planticola* in vitro, as demonstrated by Yan and colleagues (Yan et al. 2018). Specifically, *R. planticola* was shown to deglycosylate doxorubicin into its metabolites 7-deoxydoxorubicinol and 7-deoxydoxorubicinolone via a reductive deglycosylation mechanism. Moreover, doxorubicin was degraded anaerobically by *Klebsiella pneumoniae* and *E. coli* BW25113 in vitro (Yan et al. 2018). In a recent study, **5-fluorouracil** (5-FU) and **5-fluoro-2′-deoxyuridine** (FUDR) were found to act through bacterial ribonucleotide metabolism to elicit their cytotoxic effects in *Caenorhabditis elegans* (Garcia-Gonzalez et al. 2017), suggesting that bacteria in the host play an important role in the response to chemotherapeutics. Similar findings were also obtained in a different study (Scott et al. 2017). Finally, a recent study found that the anticancer immune effects of **cyclophosphamide** are modulated by the gut microbiota. Indeed, the changes induced by this chemotherapeutic on the gut microbiota stimulate the generation of a specific subset of “pathogenic” T helper 17 cells and immune responses typically associated to this medication (Viaud et al. 2013).

Interestingly, two studies have also highlighted a role for the microbiome in patients undergoing anti-programmed cell death 1 protein (PD-1) **immunotherapy** (Gopalakrishnan et al. 2018; Matson et al. 2018; Routy et al. 2018). The diversity and composition of the microbial community differed between responders and non-responders, accompanied by functional differences in gut bacteria in responders (including enrichment of anabolic pathways) (Gopalakrishnan et al. 2018). In the same study, immune profiling suggested enhanced systemic and antitumor immunity in responding patients with a favourable gut microbiome as well as in germ-free mice receiving faecal transplants from responding patients (Gopalakrishnan et al. 2018). Resistance to immunotherapy can be attributed to abnormal gut microbiome composition, according to a different study. Antibiotics administration inhibited the clinical benefit of immunotherapy in patients with cancer; moreover, faecal microbiota transplantation (FMT) from cancer patients into germ-free mice ameliorated the antitumor effect only when the donor was a responder, whereas FMT from non-responding patients failed to do so (Routy et al. 2018).

While a scarce knowledge exists on the link between the microbiome and drug absorption/metabolism, this topic assumes high clinical relevance, considering that changes in absorption and metabolism can correspond to alterations in drug efficacy and toxicity. There are no studies so far exploring the effects of microbial perturbations on psychotropic drug pharmacokinetics and more research is warranted, especially considered that several psychotropics have been shown to alter the gut microbiota composition (see Table 1). Overall, the growing evidence underlies a fascinating interaction between intestinal bacteria and drug efficacy, suggesting that precision medicine strategies should include the intestinal microbiota as a potential treatment modifier (Jobin 2018).

**Table 1:** Correlations between psychotropic compounds and microbes

*Psychotropic class*	*Psychotropic compound*	*Experimental approach*	*Details*	*Reference(s)*
**Antipsychotics**	Aripiprazole	In vivo	4-week administration in rats increase the relative abundances of *Clostridium*, *Ruminiclostridium*, *Intestinibacter* and *Eubacterium coprostanoligens*	Cussotto et al. 2018
		In humans	The microbiota communities of AAP-treated (including aripiprazole) and non-treated patients are significantly separated. The genera *Lachnospiraceae*, *Akkermansia* and *Sutterella* are differentially abundant in the two groups	Flowers et al. 2017
	Chlorpromazine	In vitro	Antimycobacterial properties	Kristiansen and Vergmann 1986; Molnar et al. 1977
			Synergistic effect in combination with certain antibiotics	Amaral et al. 1992
			Inhibits significantly the growth of *S. aureus* and *E. coli*	Ordway et al. 2002a; Amaral and Lorian 1991; Csiszar and Molnar 1992
	Fluphenazine	In vitro	Pronounced action against both Gram-positive and Gram-negative bacteria at concentrations of 20–100 μg/mL	Dastidar et al. 1995
	Olanzapine	In vitro	Completely inhibits the growth of *E. coli* NC101	Morgan et al. 2014
		In vivo	3-week administration in rats alters the microbiota profile in both males and females	Davey et al. 2012
			4-week administration in mice accelerates weight gain resulting from high-fat diet. The effect is absent under GF conditions but emerges quickly upon microbial colonisation of the gut	Morgan et al. 2014
			Coadministration with an antibiotic cocktail in female rats attenuates body weight gain, uterine fat deposition, macrophage infiltration of adipose tissue and plasma free fatty acid levels, all of which are increased by olanzapine alone	Davey et al. 2013
			Coadministration with the prebiotic B-GOS in female rats attenuates olanzapine-induced weight gain	Kao et al. 2018
		In humans	The microbiota communities of AAP-treated (including olanzapine) and non-treated patients are significantly separated. The genera *Lachnospiraceae*, *Akkermansia* and *Sutterella* are differentially abundant in the two groups	Flowers et al. 2017
			Cross-sectional study on psychiatric patients. No significant differences in microbiota composition at baseline between AAP users and non-users. Non-AAP users have increase in *Alistipes*. AAP-treated females have decreased diversity compared with non-treated females	Flowers et al. 2019
	Prochlorperazine	In vitro	Strongly inhibits *Bacillus* spp. and *Staphylococcus* spp.	Rani Basu et al. 2005
	Risperidone	In vivo	80 μg/day in female mice induces weight gain which correlates with an altered gut microbiota. Faecal transplant from risperidone-treated mice causes a 16% reduction in total resting metabolic rate in naïve recipients, attributable to suppression of non-aerobic metabolism	Bahr et al. 2015b
		In humans	The microbiota communities of AAP-treated (including risperidone) and non-treated patients are significantly separated. The genera *Lachnospiraceae*, *Akkermansia* and *Sutterella* are differentially abundant in the two groups	Flowers et al. 2017
			Chronic treatment in psychiatrically ill children increases the BMI and reduces the ratio of Bacteroidetes/Firmicutes. There is a gradual decrease in the Bacteroidetes/Firmicutes ratio over the ensuing months of treatment	Bahr et al. 2015a
	Thioridazine	In vitro	Antimicrobial activity against methicillin-susceptible *S. aureus*, vancomycin-resistant pathogenic strains of *Enterococcus* species, *Mycobacterium tuberculosis, Pseudomonas aeruginosa* and *Mycobacterium avium*	Hahn and Sohnle 2014; Ordway et al. 2002b; Wainwright et al. 1999; Amaral et al. 1996; Bettencourt et al. 2000; Ordway et al. 2003; Viveiros and Amaral 2001; Viveiros et al. 2005
	Trifluoperazine	In vitro	Antimicrobial activity against 46 of 55 strains of *S. aureus* at doses of 10–50 μg/mL. Antimicrobial against *Shigella* spp., *Vibrio cholerae* and *V. parahaemolyticus* at concentrations of 10–100 μg/mL	Mazumder et al. 2001
**Antidepressants**	Amitriptyline	In vitro	Out of 254 bacterial strains, 185 are inhibited at different doses, with *Staphylococcus* spp., *Bacillus* spp. and *Vibrio cholerae* being the most affected bacteria. Amitriptyline also inhibits both *Cryptococcus* spp. and *Candida albicans*	Mandal et al. 2010
		In vivo	At doses of 25 μg/g and 30 μg/g significantly protects mice from *Salmonella typhimurium*	Mandal et al. 2010
	Clomipramine	In vitro	Cytotoxic effects against both human protozoan parasites *Leishmania donovani* and *Leishmania major*	Zilberstein and Dwyer 1984
	Desipramine	In vitro	Effective against *Plasmodium falciparum*	Basco and Le Bras 1990; Salama and Facer 1990
	Escitalopram	In vitro	Antimicrobial effect on *E. coli*, but no effect on *L. rhamnosus*	Cussotto et al. 2018
	Fluoxetine	In vitro	Strong dose-dependent antimicrobial activity against *L. rhamnosus* and *E. coli*	Cussotto et al. 2018
		In vivo	4-week administration in rats completely inhibits the growth of *Succinivibrio* and *Prevotella* caecal taxa	Cussotto et al. 2018
	Imipramine	In vitro	Cytotoxic effects against both human protozoan parasites *Leishmania donovani* and *Leishmania major*	Zilberstein and Dwyer 1984
			Inhibits the growth of *E. coli* and *Yersinia enterocolitica* through interference with plasmid replication. It also inhibits the parasite *Giardia lamblia*	Csiszar and Molnar 1992; Molnar 1988; Weinbach et al. 1992
	Ketamine	In vitro	Antimicrobial activity against: *S. aureus*, *S. epidermidis*, *E. faecalis*, *S. pyogenes*, *P. aeruginosa* and *E. coli*, with *S. aureus* and *S. pyogenes* being the most sensitive strains	Begec et al. 2013; Gocmen et al. 2008
			Sustained antimicrobial activity in a dose-dependent manner against microorganisms in propofol, which is a strong growth-promoting factor	Begec et al. 2013
	Promethazine	In vitro	Inhibits the growth of *E. coli* and *Yersinia enterocolitica* through interference with plasmid replication	Csiszar and Molnar 1992; Molnar 1988
	Sertraline	In vitro	Potent antimicrobial against *E. coli*	Bohnert et al. 2011
			Inhibits the growth of *S. aureus*, *E. coli* and *P. aeruginosa* and also shows synergy in combination with antibiotics	Ayaz et al. 2015
			Potent antifungal activity against *Cryptococcus neoformans*, *Coccidioides immitis* and *Candida* spp.	Rossato et al. 2016; Trevino-Rangel Rde et al. 2016; Zhai et al. 2012; Paul et al. 2016, Lass-Florl et al. 2003
			Kills 97.5% of the promastigotes of *Leishmania donovani* at a dose of 30 mg/L. At the lowest concentration (3 mg/L), it induces significant loss of viability in the promastigotes (61%)	Palit and Ali 2008
**Antianxiety drugs**	Propranolol	In vitro	Inhibits the growth of *S. aureus* and *E. coli*	Kruszewska et al. 2004; Hadera et al. 2018
			Does not inhibit the growth of *S. aureus*	Jerwood and Cohen 2008
**Anticonvulsants/mood stabilisers**	Lamotrigine	In vitro	Good antibacterial activity against Gram-positive bacteria *B. subtilis*, *S. aureus* and *S. faecalis.* Inhibition of bacterial ribosome biogenesis	Qian et al. 2009; Stokes et al. 2014
	Lithium	In vivo	4-week administration in rats changes the caecal microbiome, with many genera being affected	Cussotto et al. 2018
	Valproate	In vitro	Inhibits *Mycobacterium smegmatis*	Esiobu and Hoosein 2003
		In vivo	4-week administration in rats changes the caecal microbiome, with many genera being affected	Cussotto et al. 2018
**Opioid analgesics**	Methadone	In vitro	Antimicrobial activity against *S. aureus*, *P. aeruginosa* and *S. marcescens*	Sheagren et al. 1977
		In humans	Chronic opioid use (methadone *N* = 1) in cirrhotic patients induces changes in microbiome composition, with lower relative abundance of Bacteroidaceae	Acharya et al. 2017
	Morphine	In vitro	Does not possess antimicrobial activity against any of the 10 microbial strains studied with the agar dilution method	Rosenberg and Renkonen 1985
		In vivo	Induces dysbiosis in a morphine-dependent murine model. The dysbiosis is associated to an increase in pathogenic bacteria and a decrease in communities associated with stress	Wang et al. 2018
			Intermittent or sustained opioid regimen in mice influences the gut microbiome and this is causally related to behaviours associated with opioid dependence	Lee et al. 2018
		In humans	Chronic opioid use (morphine sulphate *N* = 1) in cirrhotic patients induces changes in microbiome composition, with lower relative abundance of Bacteroidaceae	Acharya et al. 2017
	Tramadol	In vitro	Strong bactericidal activity against *E. coli* and *S. epidermidis.* Weak antimicrobial activity against *S. aureus* and *P. aeruginosa*	Tamanai-Shacoori et al. 2007
		In vivo	Subcutaneous injection in BALB/c-sensitive mice reduces the growth of *S. aureus* through enhancing phagocytes and tissue inflammation. It does not help eliminate *P. aeruginosa*	Farzam et al. 2018
		In humans	Chronic opioid use (tramadol *N* = 23) in cirrhotic patients induces changes in microbiome composition, with lower relative abundance of Bacteroidaceae	Acharya et al. 2017
**Drugs of abuse**	Cannabis	In vitro	Strong antimicrobial activity against a wide range of microorganisms	Appendino et al. 2008; Ali et al. 2018; Nissen et al. 2010
		In vivo	Modifications in the gut microbiota consequential to diet-induced obesity are prevented in mice treated chronically with THC	Cluny et al. 2015
		In humans	The microbiome of chronic marijuana users displays a *Prevotella*/*Bacteroides* ratio that is 13-fold lower than non-users	Panee et al. 2018
			A combination of THC and CBD mitigates experimental autoimmune encephalomyelitis by altering the gut microbiome	Al-Ghezi et al. 2017
	Cocaine	In vivo	Administration of antibiotics in mice induces an enhanced sensitivity to cocaine reward and an enhanced sensitivity to the locomotor-sensitising effects of repeated cocaine administration	Kiraly et al. 2016
		In humans	Cocaine users display a higher relative abundance of Bacteroidetes than non-users	Volpe et al. 2014
	Heroin	In humans	The composition and diversity of intestinal microbiota in a cohort of 50 patients with SUD (of which 52% on heroin) is significantly different from those of healthy controls. The relative abundance of *Thauera*, *Paracoccus* and *Prevotella* is significantly higher in SUDs compared to healthy participants	Xu et al. 2017
	Methamphetamine	In vivo	The gut microbiota of methamphetamine-treated rats differs from that of control rats. The faecal microbial diversity is higher in methamphetamine-treated rats. The genus *Phascolarctobacterium* is reduced and the family *Ruminococcaceae* is increased in metamphetamine-treated rats	Ning et al. 2017
		In humans	The composition and diversity of intestinal microbiota in a cohort of 50 patients with SUD (of which 30% on methamphetamine) is significantly different from those of healthy controls. The relative abundance of *Thauera*, *Paracoccus* and *Prevotella* is significantly higher in SUDs compared to healthy participants	Xu et al. 2017
**Alcohol**	*NA*	In vivo	4-week intermittent vaporised ethanol in mice alters the gut microbiota, increasing the levels of *Alistipes* and decreasing *Clostridium IV*, *Dorea* and *Coprococcus*	Peterson et al. 2017
			In a mouse model of alcoholic liver disease, Bacteroidetes and Verrucomicrobia are increased in mice fed alcohol	Yan et al. 2011
		In humans	Human alcoholics with dysbiosis have lower abundances of Bacteroidetes and higher ones of Proteobacteria	Mutlu et al. 2012
			Alcohol-dependent subjects have an increased intestinal permeability which is linked to significant microbiome alterations	de Timary et al. 2015; Keshavarzian et al. 2009; Leclercq et al. 2014
			In cirrhotic patients, the proportion of phylum Bacteroidetes is significantly reduced, whereas Proteobacteria and Fusobacteria are highly enriched compared to healthy controls. Enterobacteriaceae, Veillonellaceae and Streptococcaceae are prevalent in patients with cirrhosis at the family level	Chen et al. 2011
**Nicotine**	*NA*	In vitro	Active against *E. coli*, *P. aeruginosa* and *S. faecalis* at a dose of 2 μg/μL; and against *Listeria monocytogenes* and viridans streptococci at a dose of 10 μg/mL	Idrees Zaidi et al. 2012; Pavia et al. 2000
		In vivo	Influences the gut microbiota composition in a sex-specific manner in mice	Chi et al. 2017
		In humans	Induces profound changes in the gut microbiome, with an increase of Firmicutes and Actinobacteria and a decrease of Bacteroidetes and Proteobacteria at the phylum level. Smoking cessation induces an increase in microbial diversity	Biedermann et al. 2013
			Tobacco smokers display a higher relative abundance of *Prevotella*, lowered *Bacteroides* and lower Shannon diversity compared to controls	Stewart et al. 2018
**Xanthines**	Caffeine	In vitro	Inhibits the growth of *E. coli* and *E. faecalis*	Tatsuya and Kazunori 2013; Daglia et al. 2007
		In vivo	Consumption of 500 μL/day of coffee for three consecutive days in specific-pathogen-free mice decreases the levels of *E. coli* and *Clostridium* spp.	Tatsuya and Kazunori 2013
			Caffeine-rich Pu-erh tea remodels the intestinal dysbiosis in mice with metabolic syndrome. *Akkermansia muciniphila* and *Faecalibacterium prausnitzii* are the key gut bacterial links between the Pu-erh tea treatment and metabolic syndrome	Gao et al. 2018
			Chronic coffee consumption in diet-induced obese rats decreases the abundance of *Clostridium Cluster XI* and increases Enterobacteriaceae. SCFAs are largely increased in the coffee-fed rats	Cowan et al. 2014
			8 weeks of coffee consumption in rats does not alter the gut microbiota composition	Cowan et al. 2013
			Oral administration of 0.7 mg/kg/day caffeine for 21 days in mice decreases *Lactobacillus*	Kleber Silveira et al. 2018
		In humans	Consumption of 3 cups of coffee daily for 3 weeks in healthy volunteers increases the population of Bifidobacterium spp. In some subjects, there is a specific increase in the metabolic activity of Bifidobacterium spp.	Jaquet et al. 2009
	Theobromine	In vivo	2-week administration of cocoa’s theobromine in rats induces marked changes in gut microbiota. Rats that received a 10% cocoa-containing diet have lower counts of *E. coli*. Rats that received a 0.25% theobromine-containing diet have lower counts of *Bifidobacterium* spp., *Streptococcus* spp. and *Clostridium histolyticum*-*C*. *perfingens* group	Martín-Peláez et al. 2017
	Theophylline	In vivo	Consumption of fermented green tea, containing theophylline, is able to restore the changes in gut microbiota composition associated to diet-induced obesity in mice	Seo et al. 2015

Compounds are listed in alphabetic order

*AAP* atypical antipsychotic, *B-GOS* bimuno galactooligosaccharide, *BMI* body mass index, *CBD* cannabidiol, *GF* germ-free, *NA* not addressed, *SCFAs* short-chain fatty acids, *SUD* substance use disorders, *THC* Δ9 tetrahydrocannabinol

## Antipsychotics and gut microbiota: a general overview

Antipsychotics are drugs used for the prophylaxis and acute treatment of psychotic illnesses including schizophrenia and psychosis associated with depression and mania (Gardner et al. 2005). They also have an important role as an alternative or adjunct to benzodiazepines in the management of the acutely disturbed patient, for both tranquillisation and sedation. The common mechanism of action of all antipsychotics is to decrease brain dopamine function by blocking the dopamine D_2_ receptors (Laruelle et al. 2005).

Analysis of faecal microbiota from 76 elderly hospitalised patients showed that, among several therapeutic classes, the use of antipsychotics had a strong association with gut microbiota composition (Ticinesi et al. 2017). In a recent study, differences in faecal microbiota between patients with first-episode psychosis and healthy controls, were associated with response after up to 12 months of treatment (Schwarz et al. 2018), suggesting that the gut microbiota might be involved in treatment response. Specifically, *Lactobacillaceae* and *Bifidobacteria* were highly abundant in patients with first-episode psychosis and correlated positively with severity of psychotic symptoms and negatively with global functioning (Schwarz et al. 2018). In a tour de force in vitro screening study of more than 1000 drugs against 40 representative gut bacterial strains, it was found that 24% of human-targeting drugs inhibited the growth of at least one strain (Maier et al. 2018). Provocatively, nearly all subclasses of the chemically diverse antipsychotics targeted a significantly more similar pattern of species than expected from their chemical similarity, raising the possibility that antimicrobial action may not only manifest as side effect of antipsychotics, but also be part of their mechanism of action (Maier et al. 2018). This hypothesis should be ideally verified by assessing whether microbiome manipulations (i.e. antibiotic administration) have an effect on the efficacy of antipsychotics. Remarkably, the Maier et al.’s study provides an exhaustive justification for the dose used in the in vitro screening. The authors argue that, based on drug excretion patterns from published work, the chosen concentration of 20 μM is below the median colon concentration of the human-targeted drugs tested (Maier et al. 2018) and therefore has translational validity. Notably, in their experiment, human-targeted drugs that showed anticommensal activity had lower plasma and estimated small intestinal concentrations than ones with no such activity, suggesting that more human-targeted drugs would inhibit bacterial growth if probed at higher doses, closer to physiological concentrations. In a recent study in vitro, the antibacterial activity of antipsychotics against Gram-positive *Staphylococcus aureus* and Gram-negative *Escherichia coli*, *Pseudomonas aeruginosa*, *Klebsiella pneumoniae* and *Acinetobacter baumannii* was investigated. Phenothiazines and thioxanthenes showed differential antibacterial activity at concentrations ranging from 64 to 1024 μg/mL, which was independent of antibiotic-resistance patterns (Nehme et al. 2018). How these concentrations translate to those found in the colon following an oral administration of antipsychotics in humans is not clear and is not mentioned in the study; thus, these findings might lack translational relevance.

### Typical antipsychotics and gut microbiota

Typical (first-generation) antipsychotics were first developed in the 1950s and the first compounds to come into medical use were the phenothiazines, such as chlorpromazine. Typical antipsychotics are characterised by extrapyramidal adverse effects such as dystonia, Parkinsonian symptoms (bradykinesia, rigidity and tremor), akathisia, tardive dyskinesia, cardiovascular effects such as postural hypotension, prolactin increase and sedation (Leucht et al. 2009).

#### Evidence from in vitro studies

Studies on the interaction between typical antipsychotics and gut bacteria have been only carried out in vitro. Other approaches, such as in vivo or human observations, are missing from literature, and one reason for this gap might be more and more consideration is directed towards the new class of antipsychotics, the atypical (Skonieczna-Zydecka et al. 2018).

**Thioridazine**, a phenothiazine antipsychotic, has been shown to possess antimicrobial activity in vitro against methicillin-susceptible *S. aureus* (Hahn and Sohnle 2014; Ordway et al. 2002b), vancomycin-resistant pathogenic strains of *Enterococcus* species (Wainwright et al. 1999), *Mycobacterium tuberculosis* (Amaral et al. 1996; Bettencourt et al. 2000; Ordway et al. 2003; Viveiros and Amaral 2001), *Pseudomonas aeruginosa* and *Mycobacterium avium* (Viveiros et al. 2005). **Fluphenazine**, another typical antipsychotic, possesses pronounced action against both Gram-positive and Gram-negative bacteria at concentrations of 20–100 μg/mL (Dastidar et al. 1995). Upon investigation of the antimicrobial activity of **trifluoperazine** against 293 strains (from two Gram-positive and eight Gram-negative *genera)*, 46 of 55 strains of *S. aureus* were inhibited by doses of 10–50 μg/mL. This drug also inhibited strains of *Shigella* spp., *Vibrio cholerae* and *V. parahaemolyticus* at concentrations of 10–100 μg/mL (Mazumder et al. 2001). *Bacillus* spp. and *Staphylococcus* spp. were strongly inhibited by the antipsychotic **prochlorperazine**; while *E. coli*, *Salmonella*, *Klebsiella* and *Pseudomonas* were only moderately sensitive or resistant to the drug (Rani Basu et al. 2005). **Chlorpromazine**, another typical antipsychotic, had in vitro antimycobacterial properties (Kristiansen and Vergmann 1986; Molnar et al. 1977) and also exerted an inhibitory synergistic effect in combination with certain antibiotics (Amaral et al. 1992). Moreover, this medication has been shown to inhibit significantly the growth of *S. aureus* (Ordway et al. 2002a) and *E. coli* (Amaral and Lorian 1991; Csiszar and Molnar 1992). Keeping in mind that these data come from in vitro bacterial cultures, it is important to remark that a direct extrapolation and translation into the gut microbiome scenario is not always possible.

### Atypical antipsychotics and gut microbiota

Atypical antipsychotics act on numerous receptors and modulate several interacting transmitter systems. All atypicals (except amisulpride) exhibit greater antagonism of 5-HT_2_ receptors than of D_2_ receptors, compared with the typical agents. Atypical drugs that do antagonise dopamine D_2_ receptors appear to have affinity for those in the mesolimbic system rather than the nigrostriatal system, producing side effects of lesser degree. Clozapine and risperidone exert substantial antagonism of α_2_-adrenoceptors, while aripiprazole is a unique drug because it is a partial dopamine D_2_-receptor agonist that acts conversely as an antagonist in regions where dopamine is overactive, such as the limbic system (Bennett and Brown 2008).

#### Evidence from in vitro studies

The effect of **olanzapine** on growth of two commensal bacterial strains, *E. coli* NC101 and *Enterococcus faecalis* OGIRF was assessed in vitro across a range of supraphysiologic concentrations (280 to 560 μg/mL). Olanzapine completely inhibited the growth of *E. coli* at concentrations above 537 μg/mL, while it did not affect the growth of *E. faecalis* (Morgan et al. 2014).

#### Evidence from in vivo studies (rodents)

Most of the studies performed in vivo have been focusing on two atypical antipsychotics, olanzapine and risperidone. Administration of **olanzapine** for 3 weeks in rats was able to induce specific alterations of the microbiota profile in both males and females (Davey et al. 2012). Moreover, administration of olanzapine in mice exacerbated the weight gain induced by high-fat diet (Morgan et al. 2014). Interestingly, this effect was absent under germ-free conditions but emerged quickly upon microbial colonisation of the gut, suggesting that gut microorganisms might be necessary for the common adverse effect of olanzapine, weight gain (Morgan et al. 2014). As a proof of concept, the impact of antibiotics on olanzapine-induced weight gain was also demonstrated. Coadministration of an antibiotic cocktail in female rats treated with 2 mg/kg of olanzapine for 21 days, attenuated body weight gain, uterine fat deposition, macrophage infiltration of adipose tissue and plasma free fatty acid levels, all of which were increased by olanzapine alone (Davey et al. 2013). More recently, one last experiment has looked at microbiota changes and olanzapine administration. In this case, the prebiotic B-GOS (bimuno galactooligosaccharide) was administered to adult female Sprague-Dawley rats in coadministration with olanzapine (2-week, daily intraperitoneal injection at a dose of 10 mg/kg) and the intake of B-GOS significantly attenuated olanzapine-induced weight gain (Kao et al. 2018). Although B-GOS alone increased *Bifidobacteria* spp., and reduced species within the Firmicutes (*Coprococcus*, *Oscillibacter*, *C*. *coccoides*, *Roseburia intestinalis cluster*, *Clostridium XVIII cluster*) and Proteobacteria (*Escherichia*/*Shigella* spp.) phyla, no effects of olanzapine were observed. This is a discrepancy with other studies, maybe due to the duration and dose of olanzapine administration and/or the method of bacterial analysis. Importantly, additional studies are required to test whether the bacteria affected by B-GOS would proliferate beyond control levels with a longer duration of olanzapine administration at a clinically relevant dose. It is important to note that sex differences might play a key role in response to atypical antipsychotics and thus many studies to date have been performed in females; however, more investigations are warranted in male counterparts. The impact of **risperidone** on the gut microbiota has also been investigated in vivo. Female mice treated with risperidone at a dose of 80 μg/day over 2 months exhibited significant excess weight gain, due to reduced energy expenditure, which correlated with an altered gut microbiota (Bahr et al. 2015b). Interestingly, faecal transplant from risperidone-treated mice into naïve recipients caused a 16% reduction in total resting metabolic rate, attributable to suppression of non-aerobic metabolism (Bahr et al. 2015b). **Aripiprazole**, an atypical antipsychotic with a mode of action that is distinct from most currently available antipsychotic drugs, was able to induce marked changes in microbiota composition in rats following a 4-week treatment at 20 mg/kg/day. The relative abundance of various taxa including *Clostridium*, *Ruminiclostridium*, *Intestinibacter* and *Eubacterium coprostanoligens* was increased by aripiprazole administration (Cussotto et al. 2018).

#### Evidence from studies in humans

A recent study has looked at the association between intake of atypical antipsychotics (AAP) and gut microbiota. In a cross-sectional design study, faecal samples of more than 100 bipolar patients were collected and analysed through 16S ribosomal sequencing. Participants were divided in two groups: one group AAP-treated and one group drug-free at the time of faecal sample collection. Atypical antipsychotics included in the AAP cohort were clozapine, olanzapine, risperidone, quetiapine, asenipine, ziprasodone, lurasidone, aripiprazole, paliperidone and iloperidone. The microbiota communities of AAP-treated and non-treated patients were significantly separated, with AAP-treated females showing decreased species diversity compared to non-AAP-treated females, while males did not show significant diversity. Three specific genera, *Lachnospiraceae*, *Akkermansia* and *Sutterella* were differentially abundant in the two groups (Flowers et al. 2017). While this study provides critical insight into the AAP-mediated changes in gut microbiota, the report included no information regarding diet, which is an important environmental factor that drives the composition of gut microbiota. Moreover, the authors observed medication-specific microbiota differences, but it is not known how these translate into functional differences. In a cross-sectional cohort study on psychiatric patients, the effect of AAPs on the gut microbiota was examined. Although no significant differences in microbiota composition were detected at baseline between AAP users and non-users, non-AAP users showed an increase in the bacterial genus *Alistipes*. AAP-treated females also had decreased diversity compared with non-treated females (Flowers et al. 2019). One more study in humans has investigated the impact of **risperidone** on gut microbiota composition. In psychiatrically ill children, chronic treatment with risperidone was associated with an increase in body mass index (BMI) and a significantly lower ratio of Bacteroidetes/Firmicutes as compared with antipsychotic-naïve psychiatric controls. Moreover, a longitudinal observation revealed a gradual decrease in the Bacteroidetes/Firmicutes ratio over the ensuing months of treatment with risperidone (Bahr et al. 2015a). Although the small sample size and the fact that polypharmacy was not taken into account, this study offers preliminary evidence that the human gut microbiome is altered in patients treated chronically with risperidone.

## Antidepressants and gut microbiota: a general overview

Antidepressants are medications used to treat symptoms of depression, social anxiety disorder, seasonal affective disorder and mild chronic depression, as well as other conditions (Delgado 2004). Antidepressants can be broadly divided into four main classes: tricyclics (TCAs), selective serotonin reuptake inhibitors (SSRIs), monoamine oxidase inhibitors (MAOIs) and novel compounds, some of which are related to TCAs or SSRIs (see SNRIs, serotonin-noradrenaline reuptake inhibitors) (Bennett and Brown 2008). The mechanism of action is based on the “monoamine hypothesis”, which proposes that the main cause of depression is a deficiency of the neurotransmitters noradrenaline (NA) and serotonin (5-HT, 5-hydroxytryptamine) in the brain. However, different classes present different mechanisms of action: SSRIs prevent 5-HT reuptake, TCAs inhibit NA reuptake but effects on 5-HT reuptake vary widely and MAOIs increase the availability of NA and 5-HT by preventing their degradation in the presynaptic terminal (Fiedorowicz and Swartz 2004).

In a cohort of elderly subjects, intake of antidepressants was strongly associated with changes in gut microbiota composition (Ticinesi et al. 2017). A different population-level analysis of gut microbiome composition found that antidepressants were significantly correlated to microbiome composition (Falony et al. 2016). The field of antidepressants and gut microbiota is in constant expansion, but there is currently not sufficient knowledge on the effect that these drugs exert on the ecology of the gut microbiota. On the other side, a consistent amount of research has examined the antimicrobial activity that these compounds have against various bacterial strains in vitro. In the following sections, evidence related to all subclasses of antidepressant compounds will be taken into consideration.

### Tricyclic antidepressants and gut microbiota

#### Evidence from in vitro studies

TCAs in general inhibit NA reuptake and some compounds can also block 5-HT reuptake to a certain extent (Horn 1980). All studies to date looking at TCAs and microbiota have been performed in vitro.

**Clomipramine** and **imipramine** have been shown to possess cytotoxic effects against both human protozoan parasites *Leishmania donovani* and *Leishmania major* (Zilberstein and Dwyer 1984). Mandal and colleagues have analysed the antimicrobial activity of **Amitriptyline** hydrochloride against 253 bacterial strains (72 Gram-positive and 181 Gram-negative) and 5 fungal strains in vitro. Moreover, they carried out a mortality experiment with or without amitriptyline in mice challenged with a virulent strain of *Salmonella typhimurium* (Mandal et al. 2010). Out of 254 bacterial strains, 185 were inhibited at different doses of amitriptyline, with *Staphylococcus* spp., *Bacillus* spp. and *Vibrio cholerae* being the most affected bacteria (Mandal et al. 2010). Regarding fungal strains, amitriptyline inhibited both *Cryptococcus* spp. and *Candida albicans*. Finally, in the in vivo experiment, amitriptyline at 25 μg/g and 30 μg/g body weight significantly protected the mice from *Salmonella typhimurium* (Mandal et al. 2010). **Promethazine** and **imipramine** have been demonstrated to inhibit the growth of *E. coli* and *Yersinia enterocolitica* through interference with plasmid replication (Csiszar and Molnar 1992; Molnar 1988), and imipramine was also able to inhibit the parasite *Giardia lamblia* (Weinbach et al. 1992). **Desipramine** has been shown to be effective against *Plasmodium falciparum* (Basco and Le Bras 1990; Salama and Facer 1990).

### Selective serotonin reuptake inhibitors and gut microbiota

#### Evidence from in vitro studies

SSRIs act, as their name indicates, predominantly by preventing 5-HT reuptake, with little or no effect on NA reuptake (Stahl 1998).

SSRIs have excellent activity against *Brucellae* (Muñoz-Criado et al. 1996) and they have been shown to be synergistic in combination with antibiotics against some microorganisms such as *Corynebacterium urealyticum* (Garcia-Rodriguez et al. 1991; Munoz-Bellido et al. 1996). Interestingly, SSRIs also affect the normal physiology of some bacteria, for example they inhibit slime production in coagulase-negative staphylococci (Munoz Criado et al. 1997) and inhibit swarming in swarming species in *Proteus* (Muñoz-Criado et al. 1998). Upon analysis of the antimicrobial activity of four SSRIs against *E. coli*, **sertraline** was the most potent antimicrobial compound (Bohnert et al. 2011). Since the discovery of sertraline as a strong antimicrobial, the research has been focused mainly on this compound. Sertraline inhibits the growth of *S. aureus*, *E. coli* and *P. aeruginosa*, and it also has synergy in combination with antibiotics (Ayaz et al. 2015). Moreover, sertraline has potent antifungal activity against *Cryptococcus neoformans* (Rossato et al. 2016; Trevino-Rangel Rde et al. 2016; Zhai et al. 2012), *Coccidioides immitis* (Paul et al. 2016) and *Candida* spp. (Lass-Florl et al. 2003). In a different study, it was shown that sertraline was able to kill 97.5% of the promastigotes of *Leishmania donovani* at a dose of 30 mg/L while, at the lowest concentration (3 mg/L), it induced significant loss of viability in the promastigotes (61%) (Palit and Ali 2008). **Fluoxetine** had a strong dose-dependent antimicrobial activity in vitro against *L. rhamnosus* and *E. coli*, while **escitalopram** only exerted a minor antimicrobial effect on *E. coli*, without affecting the growth of *L. rhamnosus* (Cussotto et al. 2018).

#### Evidence from in vivo studies (rodents)

Evidence from our laboratory has recently shown that 4 weeks of fluoxetine administration in drinking water in rats at a translationally relevant dose of 10 mg/kg/day completely inhibited the growth of *Succinivibrio* and *Prevotella* caecal taxa (Cussotto et al. 2018). Whether the microbiome changes influence the efficacy and/or toxicity of fluoxetine still needs to be teased apart.

### Other antidepressants and gut microbiota

#### Evidence from in vitro studies

Interestingly, the era of antidepressants started with **isoniazid**, a compound that also has antimicrobial activity against *Mycobacterium tuberculosis* and is currently used to treat tuberculosis (Jena et al. 2014; Lei et al. 2000). **Ketamine** is a non-competitive NMDA (*N*-methyl-d-aspartate) antagonist that acts at the PCP (phencyclidine) binding site in the NMDA receptor and possess a fast onset of action as antidepressant (Bennett and Brown 2008). Ketamine showed antimicrobial activity in vitro against six different strains of bacteria: *S. aureus*, *S. epidermidis*, *E. faecalis*, *S. pyogenes*, *P. aeruginosa* and *E. coli*, with *S. aureus* and *S. pyogenes* being the most sensitive strains (Begec et al. 2013; Gocmen et al. 2008). There is currently little known regarding the effects of ketamine on gut microbiota and other classes of antidepressants, such as MAOIs (monoamine oxidase inhibitors) and SNRIs (serotonin-norepinephrine reuptake inhibitors), have not been investigated. Given the wide range of antimicrobial effects that most antidepressants show against different strains, it is perhaps not surprising to speculate that SNRIs or MAOIs might exert a microbial effect. This represents a future direction for research.

## Antianxiety drugs and gut microbiota

### Evidence from in vitro studies

The literature to date lacks comprehensive studies investigating the effects of antianxiety agents on the gut microbiome; however, some studies in vitro have been carried out to assess whether these compounds possess antimicrobial activity. **Propranolol** is a beta-receptor blocker that is commonly used to overcome the somatic symptoms of anxiety such as tachycardia and palpitations (Whitlock and Price 1974). In vitro, this compound was able to inhibit the growth of *S. aureus* (Kruszewska et al. 2004) and *E. coli* (Hadera et al. 2018). However, the data are divergent, as a different study showed that propranolol did not inhibit the growth of *S. aureus* (Jerwood and Cohen 2008). More research in vivo and in humans is warranted to investigate the microbial effects of antianxiety drugs.

## Anticonvulsants/mood stabilisers and gut microbiota

Mood stabilisers are used to treat mood disorders, characterised by intense and sustained mood shifts, typically bipolar disorder, borderline personality disorder and schizoaffective disorder. Many agents described as mood stabilisers are also categorised as anticonvulsants (Rapoport et al. 2009). No population-based studies have been carried out to date looking at the influence of anticonvulsants on the microbiome, but some preclinical data exist and are examined in the following sections.

### Evidence from in vitro studies

We have recently screened the antimicrobial activity of **lithium** and **valproate** against *E. coli* and *L. rhamnosus* in vitro and these two medications did not inhibit the growth of the two bacteria (Cussotto et al. 2018). Interestingly, however, valproate has previously been shown to inhibit *Mycobacterium smegmatis* but not to affect *E. coli* (Esiobu and Hoosein 2003). **Lamotrigine** showed good antibacterial activity against Gram-positive bacteria *B. subtilis*, *S. aureus* and *S. faecalis* (Qian et al. 2009) and inhibition of bacterial ribosome biogenesis (Stokes et al. 2014). Finally, some evidence also showed that gabapentin and topiramate possess differential antimicrobial activity in vitro (Kruszewska et al. 2004).

### Evidence from in vivo studies (rodents)

A 4-week administration of **lithium** and **valproate** in the chow of Sprague-Dawley rats was able to change markedly the caecal microbiome (Cussotto et al. 2018). Bacterial richness was increased in both treatments compared to vehicle-treated animals; moreover, at the genus level, lithium increased the relative abundance of *Ruminococcaceae* and decreased *Bacteroides*, while valproate decreased the relative abundance of *S24-7 uncultbact* and increased *Ruminococcaceae* (Cussotto et al. 2018). Valproate, but not lithium, also affected the levels of SCFAs (short-chain fatty acids) in the caecum. How these microbial changes relate to drug efficacy is not clear. Moreover, it is also not clarified whether these drugs affect directly the gut microbiota (i.e. they reach the caecum) or indirectly (i.e. through gut-brain signalling).

## Opioid analgesics and gut microbiota

Opioid analgesics act to reduce the intensity and unpleasantness of pain. They produce their effects by activating specific G protein-coupled receptors in the brain, spinal cord and peripheral nervous system (Trang et al. 2015). Acting as agonists at opioid receptors, these compounds reduce neuronal excitability and inhibit the release of pain neurotransmitters (Conlon and Bird 2015).

### Evidence from in vitro studies

**Morphine** did not possess antimicrobial activity against any of the 10 microbial strains studied with the agar dilution method (Rosenberg and Renkonen 1985). Another opioid analgesic, **tramadol**, had strong bactericidal activity in vitro against *E. coli* and *S. epidermidis* and weak antimicrobial activity against *S. aureus* and *P. aeruginosa* (Tamanai-Shacoori et al. 2007). **Methadone** exerted antimicrobial activity in vitro against *S. aureus*, *P. aeruginosa* and *S. marcescens* (Sheagren et al. 1977).

### Evidence from in vivo studies (rodents)

In a morphine-dependent murine model, significant shifts in the gut microbiome and metabolome within 1 day following **morphine** treatment were detected. Morphine was administered through the pellet implantation method, so that plasma levels of morphine were maintained in the 0.6–2.0 μg/mL range (range observed in opioid abusers and patients on opioids for moderate to severe pain). Morphine-induced alterations in gut microbial composition were associated to a significant increase in pathogenic bacteria and a decrease in communities associated with stress tolerance (Wang et al. 2018). In a different study in mice, both intermittent and sustained morphine administration influenced the gut microbiome in a way that was causally related to behaviours associated with opioid dependence (Lee et al. 2018). Interestingly, subcutaneous injections of **tramadol** reduce the growth of *S. aureus* through enhancing phagocytes and tissue inflammation; however, it does not eliminate *P. aeruginosa* (Farzam et al. 2018).

### Evidence from studies in humans

One study has examined the effect that opioids might have on the gut microbiota in humans. In a cohort of cirrhotic patients, chronic opioid use (hydromorphone *N* = 7, fentanyl *N* = 1, methadone *N* = 1, morphine sulphate *N* = 1, oxycodone *N* = 23, Percocet *N* = 3, tramadol *N* = 23 and combinations of the drugs *N* = 3) was associated to significant changes in microbiome composition, with lower relative abundance of Bacteroidaceae and Ruminococcaceae (Acharya et al. 2017). This analysis was carried out at drug class level, and it was not possible to discriminate between the effects induced by each single compound.

## Drugs of abuse, alcohol, nicotine and gut microbiota

Considering that accumulating evidence supports the role of the gut microbiota in central nervous system (CNS) function, the interaction between the gut microbiome and drugs of abuse, as well as alcohol and nicotine, represents an expanding field.

### Evidence from in vitro studies

**Ketamine** was antimicrobial in vitro in a dose-dependent manner against some microorganisms in propofol, which is a strong growth-promoting factor (Begec et al. 2013). The ketamine MIC (minimal inhibitory concentration) was 19.5 μg/mL for *S. aureus* and 312.5 μg/mL for *E. coli* and *P. aeruginosa*. As ketamine has antidepressant potential, some of its microbial effects have already been described in the section “Other antidepressants and gut microbiota”.

Cannabis is obtained from the annual plant *Cannabis sativa* and its varieties *Cannabis indica* and *Cannabis americana*. Psychological reactions to cannabis vary widely, depending on the predisposition of the individual and can include euphoria, memory impairments and time-spatial sense impairments. In vitro assays have shown that **cannabis** exerts a strong antimicrobial activity against a wide range of microorganisms (Appendino et al. 2008; M M Ali et al. 2018; Nissen et al. 2010).

Nicotine, one of the main components of tobacco, possesses all the characteristics of a drug of dependence. It modulates dopamine activity in the midbrain, particularly in the mesolimbic system, which promotes the development and maintenance of reward behaviour (Rice and Cragg 2004). Two studies in vitro have evaluated the antimicrobial activity of **nicotine**. The psychotropic compound was active against *E. coli*, *P. aeruginosa* and *S. faecalis* at a dose of 2 μg/μL (Idrees Zaidi et al. 2012) and against *Listeria monocytogenes* and *Viridans streptococci* at a dose of 10 μg/mL (Pavia et al. 2000).

### Evidence from in vivo studies (rodents)

The gut microbiota of rats that undergoing **methamphetamine**-induced conditioned place preference is different from that of control animals. Moreover, the faecal microbial diversity is slightly higher in methamphetamine-treated rats. The propionate-producing genus *Phascolarctobacterium* is attenuated in methamphetamine-treated rats and the family *Ruminococcaceae* is increased in the same group (Ning et al. 2017). In addition, the short-chain fatty acid propionate was decreased in the faecal matter of rats that received methamphetamine (Ning et al. 2017). The microbiome might play a role also in **cocaine** addiction: administration of antibiotics in mice induced an enhanced sensitivity to cocaine reward and an enhanced sensitivity to the locomotor-sensitising effects of repeated cocaine administration (Kiraly et al. 2016). Regarding **cannabis**, recent evidence has shown that modifications in the gut microbiota consequential to diet-induced obesity are prevented in mice treated chronically with *Δ*^*9*^**tetrahydrocannabinol** (THC), the major psychoactive constituent of cannabis (Cluny et al. 2015).

Alcohol generally exerts on cells in the CNS a depressant effect that is probably mediated by particular membrane ion channels and receptors (Whitlock and Price 1974). Alcohol enhances inhibitory GABA_A_-stimulated flux of chloride through receptor-gated membrane ion channels, a receptor subtype effect that might be involved in the motor impairment caused by alcohol (Abrahao et al. 2017). Exposure to 4 weeks of chronic intermittent vaporised **ethanol** in mice markedly altered the gut microbiota, increasing the levels of *Alistipes* and decreasing *Clostridium IV*, *Dorea* and *Coprococcus* (Peterson et al. 2017). In a mouse model of alcoholic liver disease, Bacteroidetes and Verrucomicrobia were increased in mice fed alcohol compared with a relative predominance of Firmicutes in control mice (Yan et al. 2011). Several other studies in rodents have highlighted a correlation between chronic alcohol consumption, leading to liver disease, and microbiome composition (Fouts et al. 2012; Guarner et al. 1997; Yan and Schnabl 2012). Interestingly, corroborating the idea that the gut microbiome might play a role in alcohol consumption, two dietary means have been used as modulators of the gut microbiome during alcohol consumption. Saturated and unsaturated dietary fats (Kirpich et al. 2016), for example, as well as rhubarb extract (Neyrinck et al. 2017), have been shown to modulate the changes in gut microbiota induced by alcohol intake.

Finally, the psychotropic **nicotine** administered in drinking water influenced the gut microbiota composition in a sex-specific manner in mice. In treated females, *Christensenellaceae*, *Anaeroplasmataceae* and unassigned families in the orders *Bacillales* were significantly reduced. Families such as *Turicibacteraceae* and *Peptococcaceae* were largely increased in male counterparts (Chi et al. 2017).

### Evidence from studies in humans

In a human cohort, **cocaine** users displayed a higher relative abundance of Bacteroidetes than non-users (Volpe et al. 2014). The composition and diversity of intestinal microbiota in a cohort of 50 patients with substance use disorders (SUD; of which 52% on **heroin** and 30% on **methamphetamine**) was significantly different from those of healthy controls. The relative abundance of *Thauera*, *Paracoccus* and *Prevotella* was significantly higher in SUD patients compared to healthy participants (Xu et al. 2017). The intestinal microbiota of SUD people would change independently of the type of substance abused, suggesting that the global switch of lifestyle due to SUD in general could be responsible for the changes in microbiome. Importantly, almost all patients with SUDs are involved in alcohol and tobacco addiction, which may also account for the microbiome effects (Xu et al. 2017). The microbiome of chronic marijuana users displayed a *Prevotella*/*Bacteroides* ratio that was 13-fold lower than the one of non-users (Panee et al. 2018). A combination of **THC** and **cannabidiol** (CBD) has been shown to mitigate experimental autoimmune encephalomyelitis (EAE) by altering the gut microbiome (Al-Ghezi et al. 2017).

Regarding **alcohol**, the mucosa-associated colonic microbiome was altered in alcoholics compared to control participants. Specifically, the alcoholics with dysbiosis had lower median abundances of Bacteroidetes and higher ones of Proteobacteria. Moreover, these alterations were correlated with high levels of serum endotoxin in a subset of the samples (Mutlu et al. 2012). Two similar studies have demonstrated that alcohol-dependent subjects have an increased intestinal permeability which is linked to significant microbiome alterations (de Timary et al. 2015; Keshavarzian et al. 2009; Leclercq et al. 2014). Bacterial overgrowth was found in the jejunum of patients with chronic alcohol abuse (Bode et al. 1984). In cirrhotic patients, the proportion of phylum Bacteroidetes was significantly reduced, whereas Proteobacteria and Fusobacteria were highly enriched compared to healthy controls. Moreover, Enterobacteriaceae, Veillonellaceae and Streptococcaceae were prevalent in patients with cirrhosis at the family level (Chen et al. 2011).

**Nicotine** consumption and also smoking cessation induced profound changes in the gut microbiome in humans, with an increase of Firmicutes and Actinobacteria and decrease of Bacteroidetes and Proteobacteria at the phylum level. In addition, smoking cessation induced an increase in microbial diversity (Biedermann et al. 2013). The effect of tobacco smoke on the oral and gut microbiome has been recently investigated in a human cohort, where tobacco smokers displayed a higher relative abundance of *Prevotella*, lowered *Bacteroides* and lower Shannon diversity in tobacco smokers compared to controls (Stewart et al. 2018).

## Xanthines and gut microbiota

The three xanthines caffeine, theophylline and theobromine occur naturally in plants. These compounds have complex and incompletely elucidated actions, which include inhibition of phosphodiesterase (the enzyme that breaks down cyclic AMP), effects on intracellular calcium distribution and noradrenergic function (Bennett and Brown 2008). All xanthines stimulate mental activity to different extents and their effects vary according to the mental state and personality of the subject (Bennett and Brown 2008).

### Evidence from in vitro studies

Xanthines were screened against several microbial strain and all compounds displayed antimicrobial activity, with caffeine being the most effective compound (Raj and Dhala 1965). The morphology of *Aerobacter aerogenes* and *A. cloacae* was affected by **caffeine** (Raj and Dhala 1965). Coffee also inhibited the growth of *E. coli* and *E. faecalis* in vitro (Tatsuya and Kazunori 2013). However, this is not the first study showing that caffeine has antimicrobial activity in vitro, as a previous experiment had already demonstrated this concept (Daglia et al. 2007).

### Evidence from in vivo studies (rodents)

A two-week administration of cocoa’s **theobromine** to healthy adult rats was shown to induce marked changes in gut microbiota composition. Specifically, rats that received a 10% cocoa-containing diet had lower intestinal counts of *E. coli*, whereas rat that received a 0.25% theobromine-containing diet had lower counts of *Bifidobacterium* spp., *Streptococcus* spp. and *Clostridium histolyticum-C. perfingens* group compared to normal-fed rats (Martín-Peláez et al. 2017). Consumption of fermented green tea, containing **theophylline**, was able to restore the changes in gut microbiota composition associated to diet-induced obesity in mice (Seo et al. 2015). In a different study, consumption of 500 μL/day of coffee for three consecutive days in specific-pathogen-free mice induced *E. coli* and *Clostridium* spp. counts to decrease significantly (Tatsuya and Kazunori 2013). **Caffeine**-rich Pu-erh tea remodelled the intestinal dysbiosis in mice with metabolic syndrome (Gao et al. 2018). Specifically, *Akkermansia muciniphila* and *Faecalibacterium prausnitzii* were speculated to be the key gut bacterial links between the Pu-erh tea treatment and metabolic syndrome at the genus and species levels (Gao et al. 2018). Chronic coffee consumption in diet-induced obese rats was accompanied by decreased abundance of *Clostridium Cluster XI* and increased levels of *Enterobacteriaceae*. Moreover, SCFAs (short-chain fatty acids) were largely increased in the coffee-fed rats (Cowan et al. 2014). It is important to note that studies on the effects of caffeine on gut microbiota are not always consistent, for example in a different experiment on rats, 8 weeks of coffee consumption did not alter the gut microbiota composition (Cowan et al. 2013). A 3-week regimen with oral administration of 0.7 mg/kg/day in mice decreased *Lactobacillus* ratios compared to controls, but none of the other taxa were affected (Kleber Silveira et al. 2018).

### Evidence from studies in humans

**Caffeine** consumption has received much attention in recent years in relation to microbiome alterations often associated to metabolic disorders. Consumption of 3 cups of coffee daily for 3 weeks in healthy volunteers did not alter faecal profiles of the dominant microbiota, but increased the population of *Bifidobacterium* spp. (Jaquet et al. 2009). Moreover, in some subjects, there was a specific increase in the metabolic activity of *Bifidobacterium* spp. (Jaquet et al. 2009).

## Conclusion

In Harry Potter, the *basilisk* hidden in the *Chamber* has the power to petrify people and poses a threat for *Hogwarts School*: what happens in the *Chamber* will affect the entire school and vice versa. Similarly, increasing evidence suggests that the gut microbiome affects and can be affected by various chemical compounds. This bidirectional influence is more and more studied in relation to psychotropic compounds. Initially thought to work only in the brain, in recent years, psychotropic compounds have shown antimicrobial activity in vitro and/or the ability to affect the gut microbiome in vivo. It is evident from the data reviewed here that different microbial effects have been attributed to psychotropic compounds, ranging from medications to drugs of abuse, caffeine and alcohol. The challenge now is to assess the functional role of these microbial changes. It is important to note that the results up to now come mainly from in vitro experiments on isolated strains and further clinical/preclinical experimentation is required to understand whether, for example drug-mediated microbial changes are complementary mechanisms of action or are responsible for the side effects associated to these compounds. Moreover, in terms of polypharmacy, more work is needed to investigate the impact of combinations of drugs on the microbiome. As our knowledge of the gut microbiome increases, the major lesson is that the *Chamber of Secrets* should be taken into account in future pharmacokinetic and pharmacodynamics analysis of known drugs and become part of the safety pharmacology of drugs in development. It is not bizarre to think that in the future microbiome measures will form part of clinical practice to investigate either the efficacy or side effects of psychotropic compounds. This field of research may also influence selection of individuals for clinical trials. Clearly it will need to be integrated, in a larger systems biology approach, to other -omics and biomarker measures in psychiatric patients.
